# Variability Measures of Positive Random Variables

**DOI:** 10.1371/journal.pone.0021998

**Published:** 2011-07-22

**Authors:** Lubomir Kostal, Petr Lansky, Ondrej Pokora

**Affiliations:** Department of Computational Neuroscience, Institute of Physiology, Academy of Sciences of the Czech Republic, Prague, Czech Republic; Genentech Inc., United States of America

## Abstract

During the stationary part of neuronal spiking response, the stimulus can be encoded in the firing rate, but also in the statistical structure of the interspike intervals. We propose and discuss two information-based measures of statistical dispersion of the interspike interval distribution, the entropy-based dispersion and Fisher information-based dispersion. The measures are compared with the frequently used concept of standard deviation. It is shown, that standard deviation is not well suited to quantify some aspects of dispersion that are often expected intuitively, such as the degree of randomness. The proposed dispersion measures are not entirely independent, although each describes the interspike intervals from a different point of view. The new methods are applied to common models of neuronal firing and to both simulated and experimental data.

## Introduction

One of the most fundamental problems in computational biology is the problem of neuronal coding, the question of how information is represented in neuronal signals [1], [2]. The discharge activity of neurons is composed of series of events called action potentials (or spikes). It is widely accepted, that information in neuronal systems is transferred by employing these spikes. The shapes and durations of individual spikes are very similar, therefore it is generally assumed that the form of the action potential is not important in information transmission. When a stimulus is presented, the responding neuron usually produces a transient response followed by a sustained one, which is often treated as stationary in time [3] . The firing rate of the sustained part of the response depends on the stimulus, however, the stimulus can be also “encoded” in the statistical structure of the interspike intervals (ISI) by the *temporal coding* [1], [4]–[6].

While the description of neuronal activity from the rate coding point of view is relatively straightforward [7] , the temporal code allows infinite number of alternatives. Spike trains with equal firing rates may turn out to be different under various measures of their statistical structure beyond the firing rate. For example, even more than a half century ago, coefficient of variation (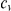) of ISIs was reported to encode information about light intensity in adapted cells of the horseshoe crab [1], [8]. Similarly, changes in the level of bursting activity, also characterized by 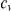, are reported to be the proper code for edge detection in certain units of visual cortex [9]. In general, the bursting nature of neuronal firing is commonly described by 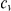 [10].

In order to describe and analyze the way information is represented in spike trains, methods for their mutual comparison are needed. Although the ISI probability density function (or histogram of data) usually provides a complete information, one needs quantitative methods [11]–[13], especially since a visual inspection of the density shape can be misleading. Here we restrict our attention to the measures of the neuronal firing precision, e.g., of the the ISI distribution dispersion. We investigate the properties of the standard deviation, the entropy-based dispersion and the Fisher information-based dispersion. Although standard deviation is used ubiquitously and is almost synonymous to the “measure of statistical dispersion”, we show, that it is not well suited to quantify some aspects of spiking activity that are often expected intuitively [4], [14]. We will show, that the diversity or randomness of ISIs is better described by entropy-based or Fisher information-based dispersions. The difference between entropy and Fisher information descriptions lies in the fact that the Fisher information describes how “smooth” is the distribution, while the entropy describes how “even” it is. The “smoothness” and “evenness” might be at first thought interchangeable, but we show that it is not the case.

The illustration of the proposed methods is provided on simple and frequently employed models of stationary neuronal activity, given by lognormal, gamma and inverse Gaussian distributions of ISIs. Finally, we apply the theory on experimental data obtained by recording the spontaneous activity of rat olfactory neurons [15].

## Methods

Statistical methods and methods of probability theory and stochastic point processes are widely applied to describe and to analyze neuronal firing [16]–[18]. The probabilistic description of spiking times results from the fact, that the positions of spikes cannot be predicted exactly, only the probability that the spike occurs is given [18]. Thus, under suitable conditions, the ISI or time-to-first spike after the stimulus onset can be described by a continuous positive random variable. We denote this random variable as 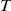. Complete description of 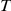 is given by its probability density function 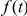, defined on 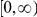.

The (statistical) dispersion is a characteristics of “variability” or “spread” of the distribution of the random variable 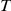. There are different dispersion measures described in the literature and employed in different contexts, e.g., standard deviation, inter-quartile range [19], mean difference [20] or the coefficient of local variance [13]. The measures have the same physical units as 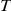.

### Standard deviation

By far, the most common measure of dispersion is the standard deviation, 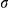, defined as the square root of the second central moment of the distribution. The corresponding *relative* dispersion measure is known as the coefficient of variation, 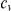,
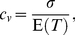
(1)where 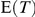 is the mean value of 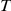. Exponential distribution implies 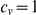, however, this values of 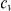 may occur for other distributions as well.

### Entropy based dispersion

The randomness of a probability distribution can be defined as the measure of “choice” of possible outcomes. Bigger choice results, intuitively, in greater randomness. For discrete probability distributions such measure of randomness is provided by the Shannon entropy, which is known to be a unique, consistent with certain natural requirements [21]. The Shannon entropy is generally infinite for continuous variables, and therefore it cannot be used for our purposes. Formally, the notion of *differential entropy*, 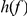, of probability density function 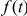, is introduced as
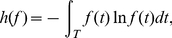
(2)however, the value 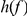 can be positive or negative and cannot be by itself used as a measure of randomness [22].

In order to obtain a properly behaving quantity, the entropy-based dispersion, 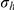, was proposed in [23],
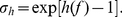
(3)The interpretation of 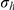 relies on the asymptotic equipartition property theorem and the entropy power concept [22]. Namely, since for the exponential probability density function 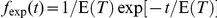 holds 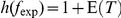 we see, that 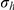 is the standard deviation of such exponential distribution, which satisfies 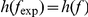. Informally, the value of 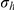 is bigger for those random variables, which generate more diverse (or unpredictable) realizations.

Analogously to Eq. (1), we define the relative entropy-based dispersion coefficient, 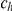, as
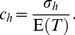
(4)Note, that Eq. (4) can be equivalently written as
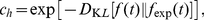
(5)where 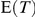 is the mean value of 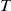 and
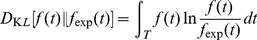
(6)is the Kullback-Leibler distance of the probability density 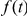 from the exponential density with the same mean as 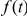. From Eq. (5) follows that 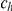 is essentially (up to the scale) equivalent to the measure of spiking randomness, 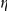, proposed in [4], since 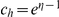.

From the properties of the Kullback-Leibler distance in Eq. (5) follows, that the maximum value of 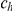 is 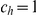, which occurs if and only if 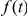 is exponential [22].

### Fisher information based dispersion

The Fisher information is a measure of the minimum error in estimating a parameter of a distribution. In a special case of the location parameter, the Fisher information 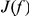 does not depend on the parameter itself, and can be expressed directly as a functional of the density 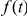 ([22], p.671),
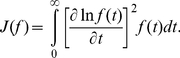
(7)We illustrate that the value of 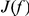 is small for smoothly-shaped probability densities. Any locally steep slope or the presence of modes in the shape of 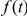 increases 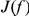 [24]. Due to the derivative in Eq. (7), certain regularity conditions are required on 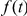. In this paper we consider only the densities for which 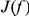 takes finite values. Further theoretical considerations are however beyond the scope of this paper.

The units of 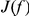 correspond to the inverse of the squared units of 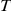, therefore we propose the Fisher information-based dispersion measure, 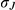, as
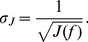
(8)In analogy with Eqns. (1) and (4) we define the relative dispersion coefficient 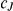 as
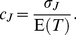
(9)For exponential distribution holds 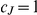, however, this value is not specific only for the case 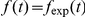.

Just as 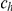 is related to the Kullback-Leibler distance by Eq. (5), we note that 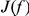 can be written as [25]

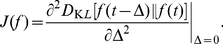
(10)Although Eq. (10) is not suitable for evaluation of 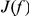, it shows, that both 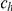 and 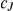 are connected on the fundamental level by the concept of the Kullback-Leibler distance.

### Basic properties of the proposed measures

**Figure 1:**
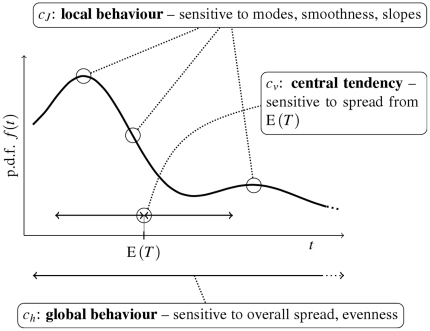
Illustration of the main features of the studied measures. Schematic example of a probability density function 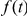 is shown. Although the evenness of the density (described by 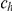) and its smoothness (described by 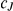) are related, the sensitivity of 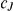 to modes and slopes enables it to differentiate shapes with otherwise equal 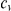 and 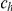.

Standard deviation (or 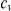) measures essentially how off-centered, with respect to 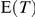, is the probability density of 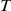 and it is sensitive to outlying values. On the other hand, 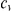 does not quantify how random, or unpredictable, are the outcomes of 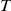. Namely, high value of 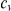 (high variability) does not indicate that the possible values of 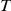 are distributed evenly [4]. On the other hand, the value of 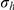 (and 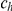) quantifies how evenly is the probability distributed over 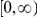. The third measure, 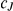, is sensitive to the modes and steepness of slopes of the density (due to the dependence on the derivative of the probability density in Eq. (7)). Since multimodal densities can be more evenly spread than unimodal ones, the behavior of 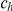 cannot be generally deduced from 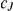 (and vice versa). The key features of the three considered dispersion measures are illustrated in Fig. 1.

**Figure 2:**
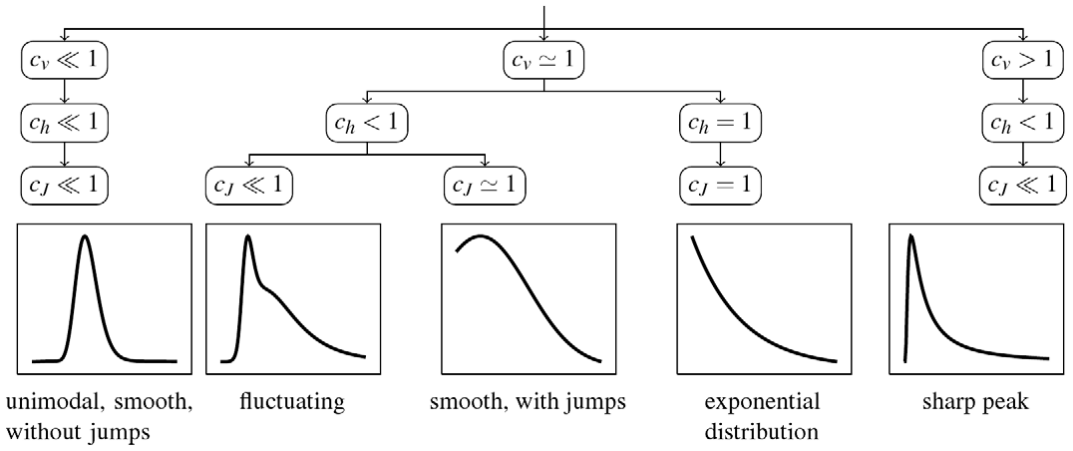
Illustration of a classification tree of probability densities based on typical values of the dispersion measures. Note, that not all combinations of values of 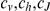 can appear. Selected identification signs or examples of corresponding distributions, which are typical but not necessarily comprehensive, are written bellow the corresponding illustrative plots of densities.

A cartoon with typical density shapes resulting from a combination of 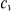, 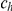 and 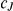 values range is shown in Fig. 2. Very small value of 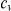 inevitably results in a density shapes concentrated around 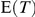, and correspondingly small values of 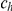 and 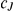. The intermediate, 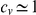, and upper range of 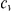 offer more variable density shapes, where 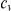 and 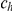 are not sufficient for their classification and 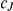 can be employed for further description. Note, that the number of possible scenarios is large and therefore Fig. 2 is not exhaustive.

## Results

### Common distributions of interspike intervals

We choose three widely employed statistical models of ISIs: gamma, inverse Gaussian and lognormal distributions, and analyze them by means of the three described dispersion coefficients 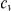, 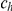 and 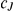.

Gamma distribution is one of the most frequent statistical descriptors of ISIs employed in analysis of experimental data [15], [26], [27]. Its probability density function parametrized by shape parameter 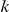 and scale parameter 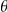 is
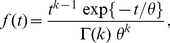
(11)where 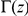 is the gamma function [28]. The mean value of the distribution is 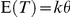 and the coefficient of variation is equal to
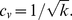
(12)For 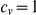, i.e. 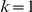, the gamma distribution becomes exponential distribution. By parametrizing the density (11) by 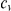 and substituting it into Eqns. (4) and (9) we obtain the entropy-based and Fisher information-based dispersion coefficients as functions of 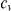,
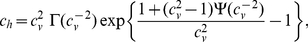
(13)

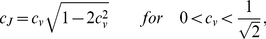
(14)where 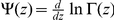 is the digamma function [28]. For details of the calculation of 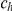 and 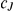 see Supporting Information S1. Note, that the gamma density is not differentiable at 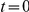 for 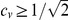, thus 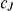 is evaluated only for 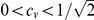.

The inverse Gaussian distribution is often used to describe neural activity and fitted to experimentally observed ISIs [26], [29], [30]. This distribution describes the spiking activity of a stochastic variant of the perfect integrate-and-fire neuronal model [18], [31]. The probability density function of the inverse Gaussian distribution parametrized by its mean, 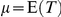, and scale parameter 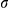 is
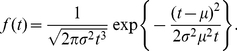
(15)The coefficient of variation is equal to
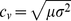
(16)and the other dispersion coefficients can be expressed as (see Supporting Information S1)
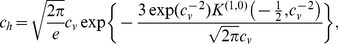
(17)

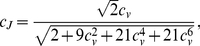
(18)where 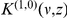 is the derivative of the modified Bessel function of the second kind, 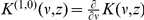 [28].

The lognormal distribution of ISIs, with some exceptions [32], is rarely presented as a result of a neuronal model. However, it represents a common descriptor in experimental data analysis [26], [30]. The lognormal probability density function parametrized by the mean, 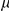, and standard deviation, 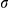, of variable 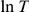 is
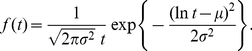
(19)In this parametrization, the mean of the lognormal distribution is 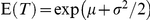 and the coefficient of variation is equal to
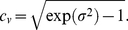
(20)The two other dispersion coefficients, expressed as functions of 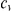, are (see Supporting Information S1)
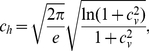
(21)

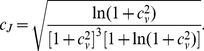
(22)


**Figure 3:**
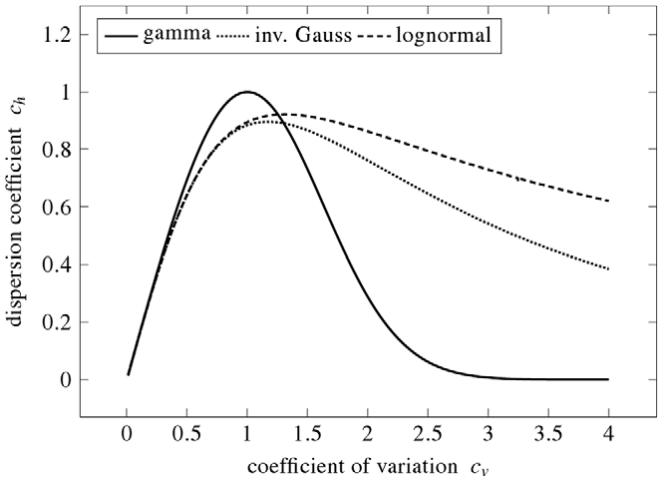
Entropy-based dispersion coefficient, 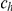, in dependence on the coefficient of variation, 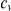. Three interspike interval models: gamma, inverse Gaussian and lognormal distribution, are employed. Both 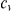 and 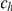 describe “spread” of the interspike intervals, but from different points of view. Coefficient of variation, 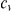, quantifies how off-centered is the mass of the probability density function, whereas 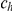 indicates how evenly is the mass distributed over all possible values. For all the shown distributions holds 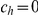 as 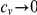 or 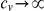.

**Figure 4:**
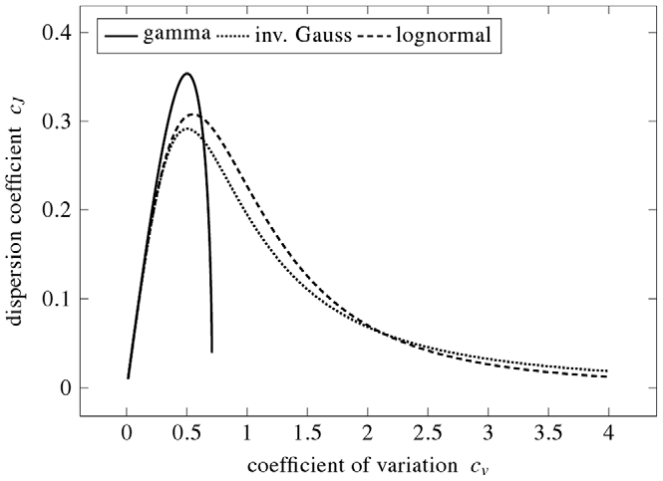
Fisher information-based dispersion coefficient, 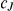, as a function of the coefficient of variation, 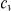, for the same distributions as in Fig. 3. The coefficient 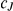 grows as the average of squared derivative of the probability density function (see Eq. (7)) becomes smaller, that means as the distribution of the interspike intervals becomes more smooth. This confirms that “smoothness” and “evenness” of the distribution (compare with Fig. 3) are different notions, although there are qualitative similarities: 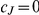 for 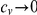 for all shown distributions, and 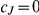 as 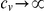 for both lognormal and inverse Gaussian distributions. Note, that dispersion coefficient 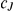 for the gamma distribution can be calculated only for 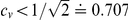.

The dependence of 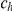 on 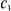 is shown in Fig. 3, the dependence of 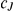 on 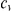 is shown in Fig. 4, for all the three mentioned distributions. Obviously, the dependencies are not linear (even not monotonous) and thus neither 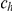 nor 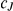 is equivalent to 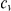. Maxima of 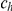 and 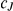 occur for different 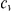 values, confirming that each of the proposed dispersion coefficients provides a different point of view. We see, that both 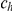 and 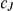 as functions of 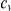 show a “
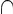
” shape with maxima around 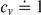 (for 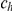) and around 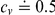 (for 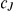). There is a reason why the maxima of 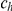 and 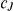 tend to occur at these values of 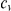. It can be shown by the methods of variational calculus, that there exists a unique distribution maximizing 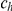: the exponential distribution for which 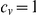. Since some densities tend to resemble the exponential density if their 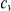 is close to 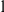, their maxima of 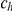 occur near this 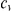 value. Similarly, there exists a unique density maximizing 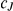; it is given in terms of the Airy functions with 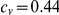. Analogously, densities with 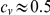 may resemble this distribution and thus attain the maximum of 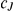 there. However, there exist distributions which does not attain the maximum of 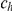 around 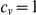 or the maximum of 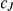 around 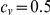. Detailed mathematical treatment of the 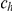
- and 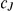-maximizing distributions is beyond the scope of the manuscript and will be published elsewhere.

Note, that the plots of 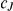 against 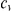 appear like a scaled version of the plots of 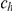 against 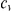, with the relative positions of the curves for each distribution preserved (to certain extent). In particular, while 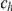 of the lognormal is always greater than 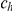 of the inverse Gaussian, the ordering is reversed for the 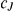 for 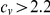.

**Figure 5:**
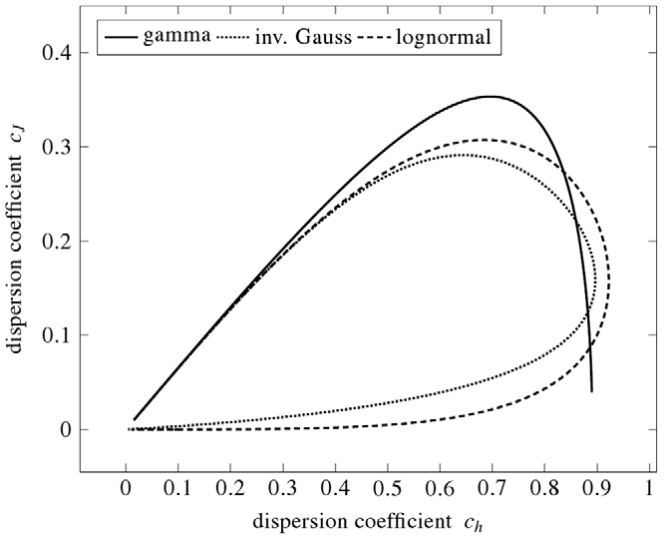
The dispersion coefficients 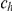 and 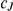 for the same distributions as in Figs. 3 and 4. The plot of dependencies between the two dispersion coefficients form closed curve for both inverse Gaussian and lognormal distribution. Starting from the origin and moving clockwise, the points on the loop correspond to the values of 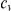 growing from 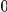 to infinity. For gamma distribution, 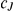 is a common unimodal function of 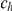.

The dependence of 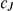 on 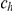 is plotted in Fig. 5. We observe, that 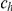 and 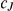 indeed do not describe the same qualities of the distribution, since a unique 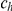 value does not correspond to a unique 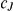 value (and vice versa). Except for the gamma distribution, the dependence between 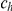 and 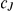 forms a closed loop, where 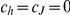 for both 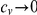 and 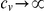.

Additionally, just as 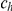 and 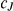 are related to 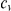 in Eqns. (13), (14), (17), (18), (21) and (22), 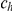 and 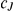 can also be related to higher statistical moments. For example, the skewness 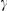 of the distribution is defined as the ratio of the third central moment and the third power of standard deviation. For gamma distribution holds 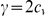, for inverse Gaussian 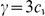 and for the lognormal 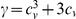. Thus the curves depicted in Fig. 3 and Fig. 4 would retain their unimodal shapes if plotted in dependence on 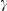.

**Figure 6:**
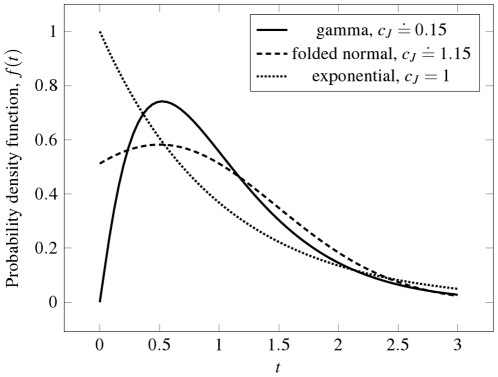
Comparison of probability density functions with 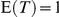, identical 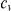 and 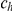 but different 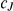. For the gamma distribution holds 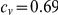, 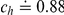 and 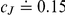. For the folded normal distribution holds 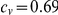, 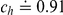 and 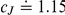. The difference between these two distributions (from the 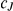 point of view) lies in the initial slope of the gamma probability density. For comparison, the exponential density (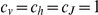) is shown.

Different distributions with equal 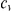 and different 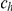 (or 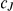) can be found, and vice versa; see Fig. 3 (or Fig. 4) for examples. Therefore, it cannot be said in general that 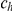, 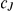 are more informative than 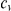. To provide an example in which 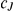 provides a different view over 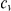 and 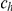, we consider the folded normal probability density with parameters 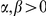


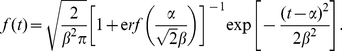
(23)The shapes of the folded normal probability density function, Eq. (23), and gamma probability density function, Eq. (11), are compared in Fig. 6 for 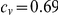. Although their values of 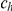 are very similar, the values of 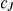 are very different. The reason lies mainly in the initial steep rise of the gamma density from zero.

### Simulated data

To illustrate the accuracy of the estimators 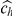 and 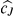 of dispersion coefficients 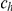 and 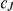, we simulated spike trains with gamma, inverse Gaussian and lognormal distributions of ISIs by employing the R and STAR software packages [26], [33]. In all the simulations the mean ISI was fixed to 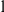, while the coefficient of variation, 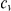, varied from 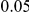 to 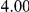 in steps of 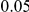. In other words, we generated random samples from the mentioned distributions with given parameters. The spike trains represented by sample point processes were constructed by using the generated values as the time intervals (ISIs) between successive events (spikes). Five thousand spike trains, each consisting of 100 ISIs, were simulated for each of the values of 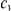 and for each of the three distributions.

In the first study, the parameters of the distributions were estimated by the maximum likelihood method. For the gamma distribution (11) the maximum likelihood estimators 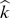 and 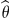 were found numerically (by minimizing the loglikelihood function). For the inverse Gaussian distribution (15) the maximum likelihood estimators were computed as
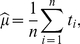
(24)

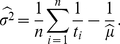
(25)Similarly, for the lognormal distribution (19) of ISIs, maximum likelihood estimators of the parameters are
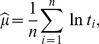
(26)

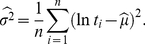
(27)


The values of coefficient of variation, 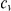, were calculated by substitution of the maximum likelihood estimates into Eqns. (12), (16) or (20). Consequently, the other two dispersion coefficients, 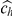 and 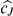, were computed by substitution of the estimated 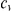 into Eqns. (13) and (14) for the gamma distribution, into Eqns. (17) and (18) for the inverse Gaussian and into Eqns. (21) and (22) for the lognormal distribution.

In the second study, the coefficient of variation was estimated by commonly used moment method as the ratio of the sample standard deviation, 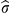, and the sample mean, 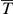,
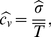
(28)for all the mentioned distributions. Both the entropy-based and Fisher information-based dispersion coefficients were then calculated by substitution of estimate (28) into the same equations for 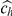 and 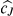 as with maximum likelihood estimates, in accordance to the respective ISI distribution.

**Figure 7:**
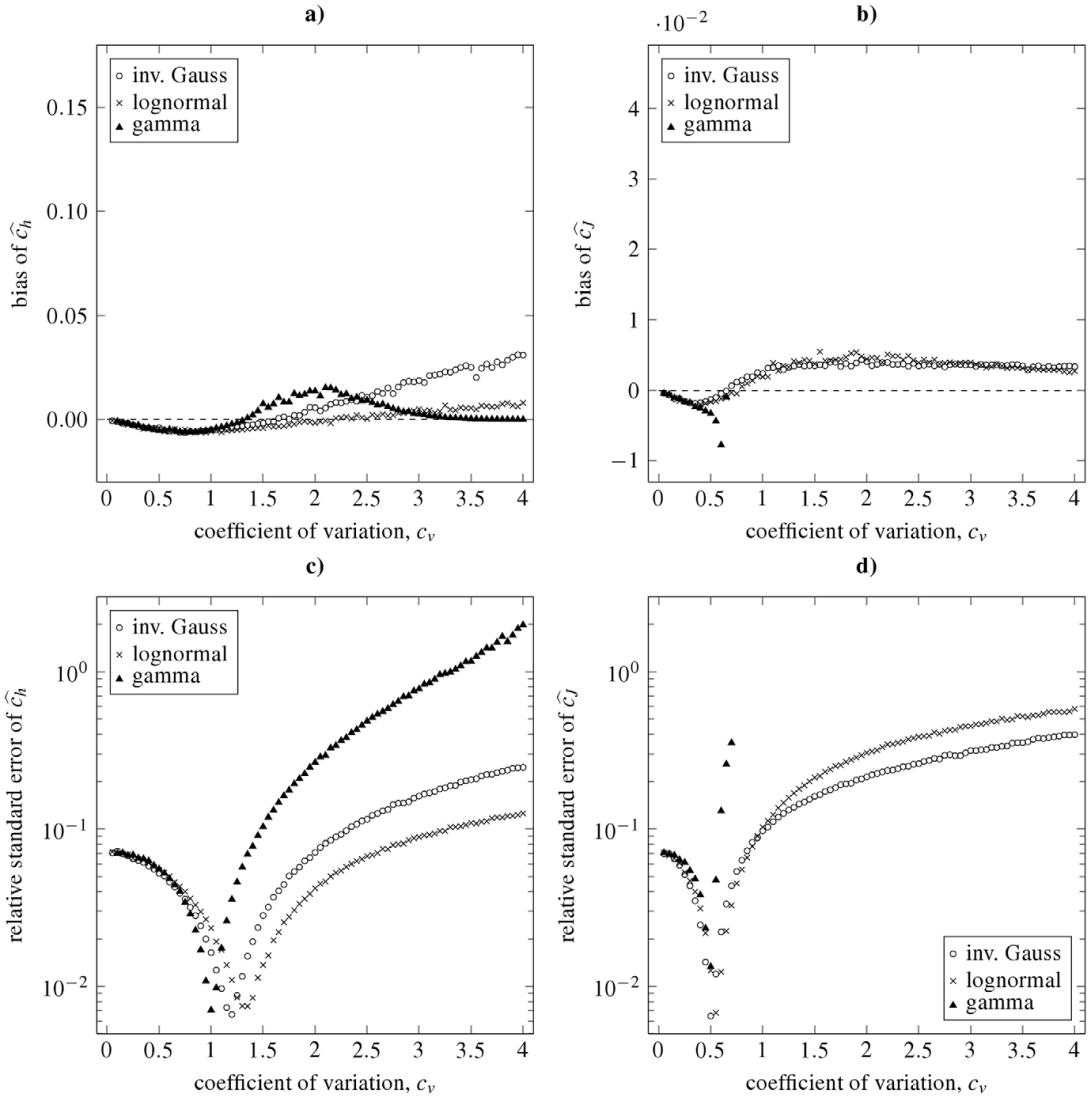
Dispersion coefficients estimation by using the maximum likelihood method from simulated data. Bias (panels **a**, **b**) and relative standard deviations (panels **c**, **d**) of the dispersion coefficients estimates 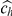 (panels **a**, **c**) and 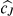 (panels **b**, **d**), in dependence on the true value of the coefficient of variation, 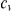, are shown. The depicted characteristics were estimated from simulated random samples drawn from inverse Gaussian (circles), lognormal (crosses) and gamma distribution (triangles). Coefficient 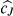 for gamma distribution (panels **b**, **d**) can be computed for 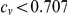 only.

**Figure 8:**
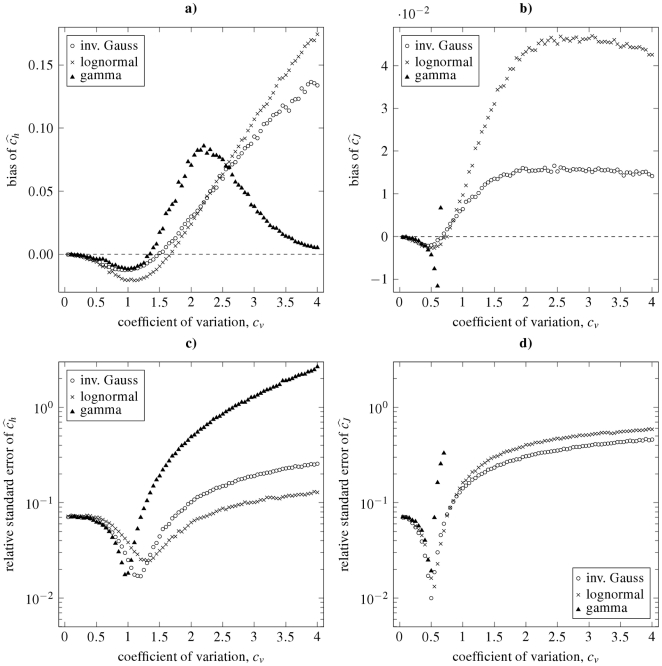
Dispersion coefficients estimation by using the moment method. The structure of the panels and the notation are equivalent to those in Fig. 7, except that the estimates 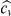, and consequently 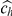 and 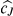 were estimated by the moment method.

The accuracy of the estimates 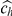 and 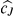 was studied for both the types of estimators. The results are depicted in Fig. 7 for the maximum likelihood estimates, and in Fig. 8 for the moment estimates. In both figures two measures of the accuracy of the estimators 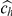 and 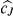 are plotted against the true values of 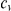 (those used for simulation). The first is the bias of the estimate, 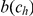, defined as
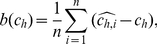
(29)where 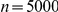 is the number of simulated spike trains and 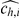 is the value estimated from the 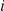-th spike train. Analogous equation is used for evaluation of 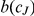. The latter measure is the relative standard error, 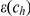, expressed as the ratio of the standard deviation and the mean value of the estimate,
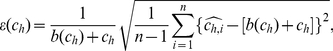
(30)and analogously for 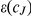. This characteristics says how accurate the values of the estimator are when calculated from random sample of given 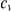. The relative standard deviation with respect to the mean value is dimensionless and therefore it is suitable for comparisons of the quality of different estimators of 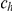 and 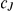.

We observe qualitative similarities in the dependencies of both the bias and the relative standard error of the estimators 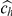 and 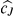. In general, we see that the estimators are biased (see panels **a**, **b** in Figs. 7 and 8), but the values of bias of the moment estimators are approximately 10 times greater than the bias of the maximum likelihood estimators. For small values of 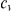 the dispersion coefficients are underestimated and the bias becomes positive as 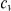 grows. For gamma distribution, the bias of 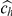 starts to decrease to zero after it attains its maxima for 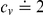, thus 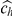 seems to be asymptotically unbiased estimator. On contrary, bias of 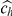 for inverse Gaussian and lognormal distribution grows as 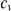 grows. There is also a difference between the maximum likelihood and moment estimator 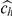: in the maximum likelihood case the bias of 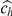 for inverse Gaussian distribution is greater than for the lognormal, the difference seems to be negligible in the case of the moment estimator.

The bias of 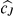 looks similar to the bias of 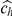 for small 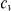. But, in contrary to 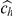, the bias of 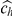 starts to decrease slowly for large values of the coefficient of variation (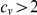). This fact can bee seen for both the inverse Gaussian and lognormal distribution. In the maximum likelihood case the bias of 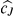 is almost the same for both these distributions. The bias of 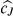 is greater for the lognormal than for inverse Gaussian distribution.

Focusing on the accuracy of the estimators (see panels **c**, **d** in Figs. 7 and 8), the shapes of the relative standard deviations of 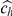 and 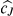 are very similar, regardless of the ISI distribution and the method used for estimation. The relative standard deviations of 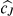 look like scaled versions of analogous characteristics of 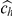. For 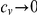 they starts at a value less than 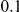.

As 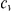 grows from zero, the relative standard deviation of the estimators decrease and attains its minima at around 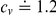 (for 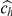) and 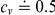 (for 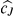), respectively. It should be noted that these minima of relative standard deviations of 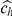 and 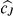 coincide with the maxima of 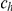 and 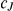 (compare with Figs. 3 and 4). In other words, the estimates 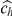 and 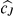 are most accurate for 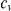 values where 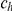 and 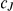 attain their theoretical maxima; but they are slightly negatively biased. For larger values of 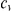 the relative standard deviations of 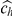 and 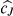 are increasing functions of 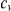. In addition, the values of relative standard deviations of the estimators for large 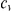 values are ordered according to the ISI distribution. The order of the estimator accuracy (from high to low) is lognormal, inverse Gaussian and gamma distribution in the case of 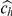, and inverse Gaussian, lognormal and gamma distribution in the case of 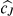.

### Experimental data

In order to examine variability or irregularity of the ISIs in real neurons using the proposed dispersion coefficients, we apply the measures on experimental data. The data come from extracellular recordings of olfactory receptor neurons of *freely breathing* and *tracheotomized* rats. Spontaneous, single-unit action potentials were recorded. The single unit nature of the recorded spikes was controlled. The experimental procedures and data analysis were published in [15], where complete details are given. The groups are not distinguishable on the basis of firing frequency only. For our purpose only samples with sufficient number of observations were chosen. Analyzed dataset consists of 6 records of ISIs from freely breathing rats and 11 records from tracheotomized rats. The sample sizes range from 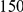 to 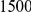 and all records were tested against nonstationarity.

**Figure 9:**
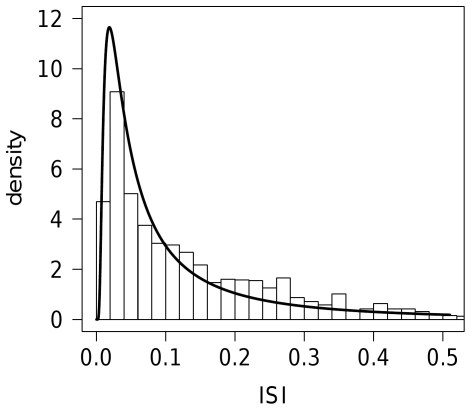
Histogram of interspike intervals from a typical record of the data. The thick curve shows the shape of probability density function fitted by the maximum likelihood method. Estimated dispersion coefficients are 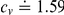, 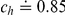 and 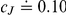.

All samples were fitted with inverse Gaussian distribution (15) as a commonly used distribution of ISI. The histogram of ISIs of typical record and fitted probability density function are depicted in Fig. 9. The mean, 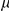, and the scale parameter 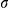 were estimated by maximum likelihood method. The fit of the data to the inverse Gaussian distribution was checked by Kolmogorov-Smirnov test. The null hypothesis was not rejected on the 5% level in any sample. The dispersion coefficients 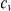, 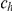 and 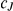 were calculated by substitution of the estimated parameters into Eqns. (16), (17) and (18).

**Figure 10:**
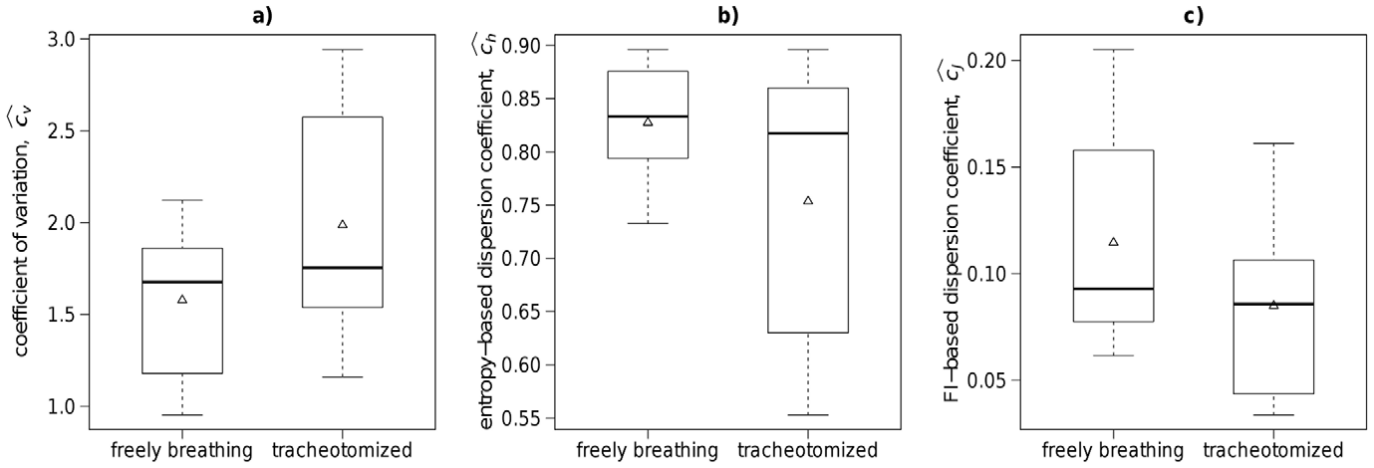
Box-and-whisker plots of estimated dispersion coefficients. The coefficients 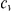 (panel **a**), 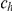 (panel **b**), and 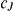 (panel **c**) estimated from the experimental data (spontaneously active rat olfactory neurons) are shown for two categories: freely breathing and tracheotomized rats. The lower and upper sides of the boxes denotes the first (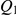) and third (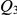) quartile, thick lines inside the boxes are medians, triangles denote mean values. The whiskers show the lowest and greatest data value between 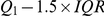 and 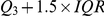 (where 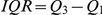 is interquartile range).

The values of estimated dispersion coefficients are summarized and shown as box-and-whisker plots in Fig. 10. Generally, the two categories, *tracheotomized* and *freely breathing*, do not differ significantly in the medians of 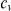, 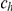 or 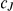. Although the ranges of the values overlap in both categories, the values of the criteria seem to be relatively specific with respect to the *freely breathing* category. The difference between mean values are greater than between medians. However, we can observe that the *tracheotomized* category achieves higher values of 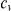 and lower values of both 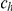 and 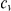 than the *freely breathing* category. Taking into account the interquartile-range and the range between the whiskers, the Fisher information-based dispersion coefficient, 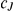, seems to be the best of the three examined coefficients to distinguish the two categories for this data. Both groups of rats were compared by employing one-sided variant of the Mann-Whitney test to the three respective dispersion coefficients. However, due to the small sample sizes, no differences between the two groups were confirmed at 95% confidence level.

**Figure 11:**
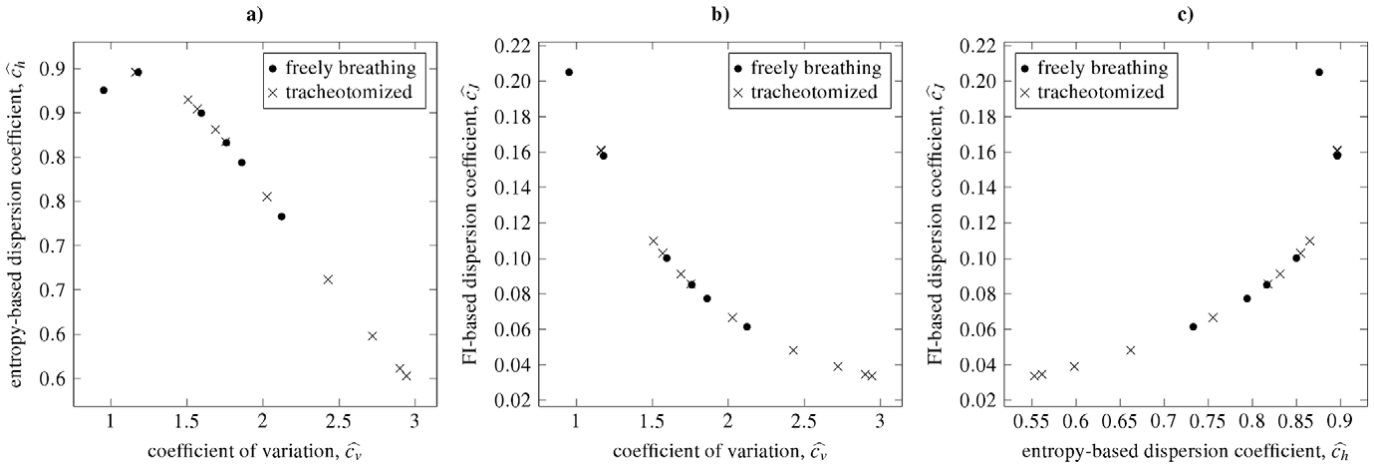
Dependencies between different dispersion coefficients for the same experimental data as in Fig. 10. The data was fitted by inverse Gaussian distribution and from maximum likelihood estimators of its parameters the dispersion coefficients were computed. Two categories of data are distinguished: tracheotomized and freely breathing rats. There are small differences in the two categories, as quantified by all the three coefficients. See Figs. 3, 4 and 5 for inverse Gaussian distribution to see the complete curves of the dependencies.

Moreover, obtained scatterplots of pairs of the dispersion coefficients 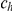 and 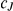 are shown in Fig. 11. The two categories of rats are best distinguishable in panel **c**), for the *tracheotomized* category having lower values of 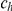 together with lower values of 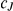 than the latter one. Note also the positions of the points in panel **c**), which confirm that there can be two different 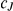 values corresponding to unique 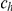 value.

## Discussion

In recent years, information-based measures of signal regularity or randomness have gained significant popularity in various branches of science [24], [34]–[37]. In this paper, we constructed dispersion-like quantities based on these information measures and applied them to the description of neuronal ISI distributions. In particular, we continued the effort initiated in [4], [23] by taking into account a variant of Fisher information, which has been employed also in different contexts [24], [38]–[41].

We are motivated by the difference between frequently mixed up notions of ISI variability and randomness, which, however, represent two different concepts. Consider, for example, a spike train consisting of “long” and “short” ISIs with no serial correlations. By adding “medium” length ISIs we do not increase the spiking variability, contrary to what expected intuitively, but decrease it. On the other hand, since the count of ISI of different lengths increases, the spiking randomness is increased. Furthermore, even if conventional analysis of two spike trains reveals no difference, the spike trains may still differ in their randomness and the difference is tractable with relatively limited amount of data [4].

Additionally, by considering the Fisher information-based dispersion coefficient, 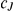, we show that ISI randomness (increasing with diversity of the ISI lengths) and probability density “smoothness” are related, but still different notions. For example, all of the tested distributions are “maximally smooth” for 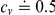 and “maximally even” (maximum ISI randomness) for 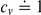.

The statistical properties of the parametric estimations of 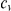 and of 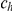 and 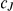 consequently, are illustrated on simulated data. The results show that the accuracy of the dispersion coefficients depends on the distribution. However, similar property can be found: estimated values of 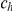 as well as of 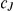 become accurate at the point of maxima of these dispersion coefficients, regardless on the used ISI distribution. It is shown that the ISI distribution as well as the method used for estimation of the parameters from the sample highly influence the bias of the estimators 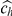 and 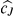.

In this paper, we used the parametrical estimates of 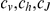 for both simulated and experimental data analyses. Specific parametric family of distributions was assumed and only the parametres were estimated. On the other hand, it is natural to ask for the non-parametric versions of the estimators. The non-parametric estimate of 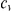 is simply calculated by using the first two sample moments. Recently [42], discussed disadvantages of this estimator, stressing out its bias. Non-parametric estimates of the entropy are known [43], [44], and we found 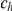 can be estimated reliably. As regards the non-parametric estimate of 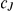, approaches based either on spline interpolation of the empirical cumulative distribution function [45] or on specialized kernel-based method for the estimation of the probability density function [46] can be used. Nevertheless, the estimation of the Fisher information-based dispersion coefficient 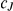 is a complex task. Preliminary results of our work in progress are promising.

The coefficients were also evaluated from the experimental data, spontaneous action potentials of olfactory receptor neurons in tracheotomized and freely breathing rats. Assuming the inverse Gaussian model, the three estimated dispersion coefficients quantify small differences in the two categories. Taking into account their variability, 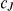 seems to be the best measure for distinguishing the categories. Other approach use the pairs of coefficients 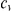, 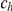 and 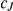 to discriminate between the groups. For the analyzed data, the pair of values 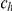 and 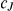 seems to be the most effective choice.

## Supporting Information

Supporting Information S1Detailed calculation of the coefficients 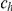 and 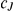 for the gamma, inverse Gaussian and lognormal distributions.(PDF)
