# Supplementary material for: Variability Measures of Positive Random Variables
Source: PLoS One. 2011 Jul 22;6(7):e21998. doi: 10.1371/journal.pone.0021998 (PMC3142115; doi:10.1371/journal.pone.0021998)
Supplement: Supporting Information S1 — Detailed calculation of the coefficients and for the gamma, inverse Gaussian and lognormal distributions. (PDF) [file pone.0021998.s001.pdf]

Variability measures of positive random variables
Supporting information S1

Lubomir Kostal[1,*], Petr Lansky[1], Ondrej Pokora[1]
1 Institute of Physiology AS CR, v.v.i., Videnska 1083, Praha 4, Czech Republic
∗ E-mail: kostal@biomed.cas.cz

Note: We refer to the equations in the main text by their number, e.g., Eq. (1), while equations in this material are numbered as Eq. (S-1), Eq. (S-2), etc.

Calculation of c_h and c_J

Gamma distribution with shape parameter k and scale parameter θ has probability density function given by Eq. (11). The differential entropy (2) of this distribution is equal to [1, p.661]

$$h(f) = \ln\theta + \ln\Gamma(k) + (1-k)\Psi(k) + k \ , \tag{S-1}$$

where $\Psi(z) = \frac{d}{dz}\ln\Gamma(z)$ is the digamma function [2]. Then, using Eqns. (3) and (4) and relation $\mathrm{E}\,(T) = k\,\theta$, the entropy-based dispersion coefficient is directly calculated,

$$c_h = \frac{\sigma_h}{\mathrm{E}\,(T)} = \frac{\Gamma(k)}{k}\exp\left\{k + (1-k)\Psi(k) - 1\right\} \ . \tag{S-2}$$

By substitution $k = 1/c_v^2$, c_h can be expressed in terms of the coefficient of variation, c_v, as

$$c_h = \Gamma(1/c_v^2)\, c_v^2\, \exp\left\{\frac{(1-c_v^2)\left[1-\Psi(1/c_v^2)\right]}{c_v^2}\right\} \ . \tag{S-3}$$

The log-density of the gamma distribution is equal to

$$\ln f(t) = (k-1)\ln t - \frac{t}{\theta} - \ln\Gamma(k) - k\ln\theta \tag{S-4}$$

and its derivative has simple expression

$$\frac{\partial \ln f(t)}{\partial t} = \frac{\theta(k-1) - t}{\theta\, t} \ . \tag{S-5}$$

The Fisher information (7) becomes

$$J(f) = \int_0^\infty \left[\frac{(k-1)^2}{t^2} - 2(k-1) + \frac{1}{\theta^2}\right] \frac{t^{k-1}\exp(-t/\theta)}{\Gamma(k)\ \theta^k}\, dt \ . \tag{S-6}$$

To calculate this integral, it is convenient to divide the expression into three integrals and employ the relations

$$\int_0^\infty t^{k-l}\exp(-t/\theta)\, dt = \theta^{k-l+1}\,\Gamma(k-l+1) \quad \text{for} \quad l = 3, 2, 1 \ . \tag{S-7}$$

In this case, the resulting Fisher information evaluates to

$$J(f) = \frac{1}{\theta^2\,(k-2)} \tag{S-8}$$

provided $k > 2$, otherwise the integral does not converge. Substituting $J(f)$ into Eqns. (8) and (9), the Fisher information-based dispersion coefficient is obtained,

$$c_J = \frac{\sqrt{k-2}}{k} \quad . \tag{S-9}$$

In the parametrization in terms of c_v, it can be expressed as relation

$$c_J = \sqrt{c_v^2(1-2c_v^2)} \ , \tag{S-10}$$

which is valid for $c_v < \sqrt{2}/2$.

Inverse Gaussian distribution with mean μ and scale parameter σ has probability density function $f(t)$ given by Eq. (15). Its log-density evaluates to

$$\ln f(t) = -\frac{1}{2}\ln\left(2\,\pi\,\sigma^2\,t^3\right) - \frac{(t-\mu)^2}{2\,\mu^2\,\sigma^2\,t} \quad . \tag{S-11}$$

Substitution into Eq. (2) gives the following expression for differential entropy. Is is convenient to separate it into several integrals in accordance with the power of variable t,

$$\begin{aligned} h(f) = \frac{1}{2}\ln(2\,\pi\,\sigma^2)\int_0^\infty f(t)\,dt\,+ \\ +\frac{3}{2}\int_0^\infty f(t)\ln t\,dt + \frac{1}{2\,\mu^2\,\sigma^2}\int_0^\infty f(t)\,t\,dt\,+ \\ +\frac{1}{2\,\sigma^2}\int_0^\infty \frac{f(t)}{t}\,dt - \frac{1}{\mu\,\sigma^2}\int_0^\infty f(t)\,dt \quad . \end{aligned} \tag{S-12}$$

All the integrals in (S-12) can be directly calculated, except the second one which needs special treatment. By [3], the density $f(t)$ can be written in terms of the modified Bessel function of the second kind, $K(\nu, z)$, with $\nu = -1/2$,

$$f(t) = \frac{1}{2\,K\left(-\frac{1}{2}, \frac{1}{\mu\,\sigma^2}\right)}\sqrt{\frac{\mu}{t^3}}\exp\left(-\frac{\mu^2+t^2}{2\,\mu^2\,\sigma^2\,t}\right) \quad . \tag{S-13}$$

The advantage of such a representation lies in the calculation of the integral

$$\int_0^\infty f(t)\ln t\,dt = \ln\mu - \sqrt{\frac{2}{\pi\,\mu\,\sigma^2}}\exp\left(\tfrac{1}{\mu\,\sigma^2}\right)K^{(1,0)}\left(-\tfrac{1}{2}, \tfrac{1}{\mu\,\sigma^2}\right) \quad , \tag{S-14}$$

which employs per partes only. Here, $K^{(1,0)}(\nu, z) = \frac{\partial}{\partial\nu}K(\nu, z)$ denotes the derivative with respect to the first argument. After substitution $\mu\,\sigma^2 = c_v^2$ for parametrization by c_v, the resulting expression for the entropy becomes

$$h(f) = \ln\mu + \frac{1}{2} + \frac{1}{2}\ln(2\,\pi\,c_v^2) - \frac{3\,\exp(1/c_v^2)}{\sqrt{2\,\pi\,c_v^2}}\,K^{(1,0)}\left(-\tfrac{1}{2}, \tfrac{1}{c_v^2}\right) \tag{S-15}$$

and the corresponding dispersion coefficient (Eqns. (3) and (4)) is equal to

$$c_h = \frac{\sigma_h}{\mathrm{E}\,(T)} = \sqrt{\frac{2\,\pi\,c_v^2}{e}}\exp\left\{-\frac{3\,\exp(1/c_v^2)}{\sqrt{2\,\pi\,c_v^2}}K^{(1,0)}\left(-\tfrac{1}{2}, \tfrac{1}{c_v^2}\right)\right\} \quad . \tag{S-16}$$

The derivative of the log-density $\ln f(t)$ is equal to

$$\frac{\partial\ln f(t)}{\partial\,t} = \frac{1}{2\,\mu^2\,\sigma^2\,t^2}\left(\mu^2 - t^2 - 3\,\mu^2\,\sigma^2\,t\right). \tag{S-17}$$

By direct computation in terms of c_v, the Fisher information can be calculated as

$$J(f) = \int_0^{\infty} \left(\frac{\partial \ln f(t)}{\partial t} \right)^2 f(t)\, dt = \frac{2 + 9c_v^2 + 21c_v^2 + 21c_v^6}{2\, \mu^2\, c_v^2} \quad . \tag{S-18}$$

Hence, employing Eqns. (8) and (9), the Fisher information-dispersion coefficient results in relation

$$c_J = \sqrt{\frac{2\, c_v^2}{2 + 9c_v^2 + 21c_v^2 + 21c_v^6}} \quad . \tag{S-19}$$

The probability density function, $f(t)$, of the lognormal distribution with parameters μ and σ is given by Eq. (19). By [1, p.662], differential entropy of the distribution is equal to

$$h(f) = \mu + \frac{1}{2} \ln(2\, \pi\, e\, \sigma^2) \quad . \tag{S-20}$$

Hence, employing Eqns. (3) and (4) and relation $\mathrm{E}\,(T) = \exp\left(\mu + \sigma^2/2\right)$, the entropy-based dispersion coefficient is evaluated as

$$c_h = \frac{\sigma_h}{\mathrm{E}\,(T)} = \sqrt{\frac{2\, \pi\, \sigma^2}{\exp(1 + \sigma^2)}} \quad . \tag{S-21}$$

In the parametrization by the coefficient of variation, c_v, by substituting $\sigma^2 = \ln(1 + c_v^2)$, it changes to

$$c_h = \sqrt{\frac{2\, \pi}{e}} \sqrt{\frac{\ln(1 + c_v^2)}{1 + c_v^2}} \quad . \tag{S-22}$$

The derivative of the logarithm of the density $f(t)$ is equal to

$$\frac{\partial \ln f(t)}{\partial t} = \frac{\ln t - \mu + \sigma^2}{\sigma^2\, t} \quad . \tag{S-23}$$

Direct calculation of the Fisher information gives

$$J(f) = \int_0^{\infty} \left(\frac{\partial \ln f(t)}{\partial t} \right)^2 f(t)\, dt = \left(1 + \frac{1}{\sigma^2} \right) \exp\left(-2\, \mu + 2\, \sigma^2 \right) \quad . \tag{S-24}$$

After substitution into Eqns. (8) and (9), expressions for the Fisher information-based dispersion coefficient are obtained:

$$c_J = \sqrt{\left(1 - \frac{1}{1 + \sigma^2} \right) \exp\left(-\frac{3}{2} \sigma^2 \right)} \tag{S-25}$$

for the μ, σ parametrization, and

$$c_J = \sqrt{\left(1 - \frac{1}{\ln(1 + c_v^2)} \right) \frac{1}{(1 + c_v^2)^3}} \tag{S-26}$$

for the c_v parametrization.

References

1. Cover TM, Thomas JA (1991) Elements of Information Theory. New York: John Wiley and Sons, Inc.

2. Abramowitz M, Stegun IA (1965) Handbook of Mathematical Functions, With Formulas, Graphs, and Mathematical Tables. New York: Dover.

3. Kawamura T, Iwase K (2003) Characterization of the distributions of power inverse gaussian and others based on the entropy maximization principle. J Japan Statist Soc 33: 95–104.
